# Diabetes and tumor risk: a 23-year Danish national cohort study

**DOI:** 10.3389/fendo.2025.1725065

**Published:** 2026-01-07

**Authors:** Katja F. Skovbjerg, Julie C. Antvorskov, Lonny M. Stokholm, Tine D. Bille, Nis Andersen, Jens Andresen, Toke Bek, Javad Hajar, Ryo Kawasaki, Caroline S. Laugesen, Sören Möller, Frederik N. Pedersen, Katja C. Schielke, Anne S. Thykjær Petersen, Flemming Pociot, Jakob Grauslund, Steffen Heegaard

**Affiliations:** 1 Department of Clinical Research, Steno Diabetes Center Copenhagen, Herlev, Denmark; 2 Department of Clinical Research, University of Southern Denmark, Odense, Denmark; 3 Open Patient Data Explorative Network, Odense University Hospital, Odense, Denmark; 4 Organization of Danish Practicing Ophthalmologists, Copenhagen, Denmark; 5 Department of Ophthalmology, Aarhus University Hospital, Aarhus, Denmark; 6 Department of Ophthalmology, Rigshospitalet Glostrup, Glostrup, Denmark; 7 Division of Public Health, Department of Social Medicine, Graduate School of Medicine, Osaka University Hospital, Osaka, Japan; 8 Department of Ophthalmology, Zealand University Hospital Roskilde, Roskilde, Denmark; 9 Department of Ophthalmology, Odense University Hospital, Odense, Denmark; 10 Department of Ophthalmology, Aalborg University Hospital, Aalborg, Denmark; 11 Steno Diabetes Center Odense, Odense University Hospital, Odense, Denmark; 12 Department of Clinical Pharmacology, Odense University Hospital, Odense, Denmark; 13 Faculty of Health and Medical Sciences, University of Copenhagen, Copenhagen, Denmark; 14 Department of Pathology, Rigshospitalet, Copenhagen, Denmark; 15 Department of Ophthalmology, Rigshospitalet, Copenhagen, Denmark; 16 Department of Clinical Medicine, University of Copenhagen, Copenhagen, Denmark

**Keywords:** cancer, diabetes, diabetes mellitus, neoplasm, pathology, T1DM, T2DM, tumor

## Abstract

**Introduction:**

Diabetes mellitus is a recognized risk factor for cancer, yet the relationship between diabetes type and tumor risk remains unclear. This study aimed to estimate the overall tumor burden, including benign, premalignant, and malignant tumors, in individuals with type 1 and type 2 diabetes.

**Methods:**

In this nationwide cohort study spanning 23 years (1999–2022), data on diabetes diagnosis, tumor development, and potential confounding variables were retrieved from multiple Danish national health registries. The cohort included more than 6.5 million individuals and 128,647 tumor events among individuals with diabetes. Crude and adjusted hazard ratios (HRs) were estimated using Cox regression.

**Results:**

For individuals with type 1 diabetes, adjusted HRs indicated no association for overall tumor development compared to individuals without diabetes. For individuals with type 2 diabetes, adjusted HRs suggested a slightly decreased hazard for overall tumor development compared to individuals without diabetes. When excluding tumors in the skin, the association between type 1 and type 2 diabetes and overall tumor development, suggested an increased hazard compared with individuals without diabetes. Our exploratory sub-analyses were stratified by tumor topography based on Systematized Nomenclature of Medicine (SNOMED) codes. Among individuals with type 1 diabetes, eight of 28 tumor groups showed reduced hazard, including the pancreas, bile ducts, and kidney. For type 2 diabetes, one group showed reduced hazard, while 22 groups, including the heart, blood vessels, and liver, showed increased hazard. Estimates from exploratory analyses should be interpreted with caution.

**Discussion:**

Our findings provide population-level evidence that advances our understanding of the possible complex metabolic links between diabetes and tumor development. Further exploration of SNOMED-based tumor classifications in future studies may provide valuable knowledge on pathological differences and refine future tumor and cancer surveillance strategies in individuals with diabetes.

## 1 Introduction

Diabetes mellitus is a prevalent endocrine disorder characterized by hyperglycemia. According to the World Health Organization, diabetes mellitus was the eighth leading cause of death globally in 2021 (1). The underlying etiology and pathophysiology classify diabetes into type 1 diabetes, an autoimmune disease characterized by beta-cell destruction, and type 2 diabetes, which is caused by the inability of cells to respond properly to the actions of insulin due to insulin resistance (2).

Epidemiological studies and meta-analysis suggest that patients with diabetes, both type 1 and type 2, are at an increased risk of developing various cancers, while at the same time, decreased risk has also been observed for certain cancer types (3–12). The extent of risk varies depending on the type of diabetes, the type of cancer, sex, and study design. However, numerous studies report inconsistent findings and a high risk of potential bias, suggesting the need for further research and clarification regarding the association between diabetes and malignant tumors (3, 4, 10, 13). Conversely, only a very limited number of studies have explored the potential relationship between diabetes and benign tumors, with observations indicating both increased and decreased risks (14–20).

The emphasis on malignant tumors in previous studies, presumably due to more severe health issues, may have limited our understanding of tumor development and prevented the possibility of fully understanding the underlying mechanisms behind the association between diabetes and the development of tumors (21). Furthermore, benign or pre-malignant tumors may still pose significant health risks, such as mechanical obstruction or other complications. In this study, we aim to address these gaps by examining the association between type 1 and type 2 diabetes and tumor development, including benign, pre-malignant, and malignant tumors.

## 2 Materials and methods

### 2.1 Study design and participants

This register-based cohort study used data from multiple Danish registries and covered the entire Danish population over a 23-year period, from January 1^st^, 1999, until December 31^st^, 2022. All individuals residing in Denmark, alive and free of any tumor diagnosis at the start of follow-up, were included, as were individuals who reached 18 years during the study period. All individuals were followed from the age of 18 years until tumor diagnosis, death, emigration, or end of follow-up, whichever came first. Participants with a tumor diagnosis before study entry were excluded from statistical analysis but included in the descriptive data.

### 2.2 Data sources

The Danish National Patient Registry provided information on all patients treated at Danish hospitals regarding the diagnosis of diabetes, based on the International Classification of Disease 10^th^ revision coding system (ICD-10) (22). The Danish National Prescription Registry, a pharmacological register, provided data on prescribed medication using Anatomical Therapeutic Chemical (ATC) classification. Data on the following ATC codes were drawn (* denotes inclusion of all subcodes): A10B* (blood glucose-lowering medication, excluding insulin), A10A* (insulin and analogues), C03*, C07*, C08*, C09* (anti-hypertensive medication), and C10* (cholesterol-lowering medication) (23). Patobank, a Danish nationwide pathology database, provided information on tumors in the form of SNOMED codes (24). The full code list, regarding above mentioned codes, can be found in **Supplementary Material (Appendix 2)**. The Danish Civil Registration System enabled linkage of individual data across these registries, through unique Personal Identification Numbers (PINs) (25). Date of birth, sex (male or female), marital status, and vital status were also drawn for the Civil Registration System. Most registers used in this study have been described in detail by Grauslund et al., 2020 (26).

### 2.3 Diabetes

Diabetes patients were divided into groups of type 1 and type 2 diabetes based on ICD-10 codes and the use of insulin or non-insulin diabetes medication. The classification of diabetes type used in this study has been developed by the Ocular And Systemic complications In diabetic retinopathy (OASIS) study group, to be used for Danish register-based studies (27). For specified definitions, see **Supplementary Material (Appendix 1)**. The reference groups were individuals without diabetes. The onset of diabetes was defined as the earliest of either the first date of diagnosis or the first date of purchase of diabetes medication, registered in the registers.

### 2.4 Tumor

In Denmark, tumors are systematically registered and classified according to pathology using SNOMED codes. In the present study, all tumors were defined based on biopsy-verified diagnoses, enabling the integration of detailed pathological information with each diagnosis. Tumor was defined as the first primary tumor occurrence after the study entry, registered in Patobank. The Danish version of SNOMED codes are based on the second edition of the College of American Pathologists version and follows the general principles of classification (24). The SNOMED codes consist of 6 axes: Topography (T), morphology (M), etiology (Æ), function (F), sickness (S), and procedure (P). All axes are followed by 5 numbers. Every pathology diagnosis consists of at a minimum M- and T-codes (24). Officially updated SNOMED codes can be found on the website of Patobank (28). In the present study all M8- and M9-codes (codes defining a tumor) were analyzed and tumors were stratified based on the exact anatomical location of the tumor, through T codes, into the following 28 groups: “Skin”, “Breast”, “Bone marrow”, “Spleen, lymph nodes, lymph vessels and thymus”, “Blood”, “Bone and joints”, “Muscle, tendon and soft tissue”, “Upper respiratory tract”, “Lower respiratory tract”, “Heart”, “Blood vessels”, “Mouth and salivary glands”, “Liver”, “Pancreas and bile ducts”, “Pharynx and tonsils”, “Esophagus and gastrointestinal tract”, “Kidney”, “Urinary tract”, “Male genitals”, “Lower female genitals”, “Upper female genitals”, “Placenta and fetus”, “Pituitary gland and pineal gland”, “Adrenal gland, glomus and paraganglia”, “Thyroid gland and parathyroid glands”, “Brain and nervous system”, “Eye” and lastly “Ear”. For exact T codes related to each topography group, see **Supplementary Material (Appendix 2)**. If a tumor group was only represented by 1–4 events, data were not available due to the national requirement for data protection and anonymity, and therefore, data will appear as “<5” in the tables.

### 2.5 Confounders

Possibly confounding variables accounted for in this study were attained age at entry, marital status, and medication use. Marital status was categorized into three groups; never married, married/living together, or divorced/widowed at the time of entry. Medication use was defined as anti-hypertensive medication use, and cholesterol-lowering medication use, based on ATC codes (specified in the “data sources” section and in **Supplementary Material, Appendix 2)**, and defined as the date of the first redeemed prescription. Medication use was thereby adjusted for as a time-varying confounder.

### 2.6 Statistical analyses

Continuous variables were presented as medians, with interquartile ranges (IQRs), and categorical variables as counts and column percentages.

For all analyses, to examine the association between type 1 and type 2 diabetes and general tumor development, including benign, pre-malignant, and malignant tumors, we presented event count and events pr. 100,000 person-years, and estimated crude and adjusted Hazard ratios (HRs) for tumor development as a binary outcome, along with 95% confidence intervals (CI). Attained age was used as the underlying time scale. CI excluding one and *p*-values below 0.05 were considered statistically significant. Because diabetes status could change during follow-up, diabetes was modeled as a time-varying exposure.

For the main analysis, we examined the association between type 1 and 2 diabetes and overall tumor development. We estimated crude HRs and adjusted HRs, adjusted for the above-mentioned confounding variables. All HRs were stratified by sex and attained age at diabetes onset, categorized as above or below 50 years, based on results from interaction analyses. Interaction analyses were performed regarding sex and age, resulting in *p*-value < 0.001 by sex and *p*-value < 0.001 by age, for type 1 diabetes, and *p*-value < 0.01 by sex and *p*-value = 0.818 by age, for type 2 diabetes.

We then performed an exploratory topography-stratified sub-analyses, estimating crude HRs and adjusted HRs, adjusted for the above-mentioned confounding variables. Each analysis included the first occurrence of a tumor within that specific topography group. Consequently, individuals could contribute to multiple sub-analyses if they developed tumors in different locations. All sub-analyses were stratified by sex.

Based on findings from our main and exploratory sub-analyses, we conducted a supplementary analysis on overall tumor development, excluding tumors in the skin. We estimated crude HRs and adjusted HRs, adjusted for the above-mentioned confounding variables. All HRs were stratified by sex and attained age at diabetes onset, categorized as above or below 50 years.

Stata version 18.0 (StataCorp LLC, College Station, TX, USA) was used for all statistics.

## 3 Results

The cohort included a total of 6,559,315 individuals, of whom 22,481 had type 1 diabetes, and 78,738 had type 2 diabetes. Across the study period, 128,647 tumor events occurred among individuals with diabetes, including 10,869 events in those with type 1 diabetes, and 117,778 events in those with type 2 diabetes (**Table 1**).

**Table 1:** Baseline characteristics at entry time when stratified into groups of type 1 diabetes (T1DM), type 2 diabetes (T2DM), and no diabetes.

Characteristic	Category	No diabetes N=6,458,096	T1DM N=22,481	T2DM N=78,738	Total N=6,559,315
Attained age at entry (years)	Median (IQR)	31 (18-51)	51 (25-67)	65 (54-75)	32 (18-52)
Marital status	Never married	3,580,765 (55.4%)	8,553 (38.0%)	9,413 (12.0%)	3,598,731 (54.9%)
	Married or living together	2,171,249 (33.6%)	9,071 (40.3%)	42,462 (53.9%)	2,222,782 (33.9%)
	Divorced or widowed	706,082 (10.9%)	4,857 (21.6%)	26,863 (34.1%)	737,802 (11.2%)
Sex	Males	3,213,996 (49.8%)	12,562 (55.9%)	41,958 (53.3%)	3,268,516 (49.8%)
	Females	3,244,100 (50.2%)	9,919 (44.1%)	36,780 (46.7%)	3,290,799 (50.2%)
Insulin use	Yes	0 (0.0%)	17,956 (79.9%)	20,605 (26.2%)	38,561 (0.6%)
Non-insulin use	Yes	1,814 (0.0%)	7,409 (33.0%)	57,814 (73.4%)	67,037 (1.0%)
Cholesterol-lowering medication use	Yes	51,519 (0.8%)	928 (4.1%)	5,348 (6.8%)	57,795 (0.9%)
Anti-hypertensive medication use	Yes	759,883 (11.8%)	9,322 (41.5%)	50,831 (64.6%)	820,036 (12.5%)

Results are given in counts (n) or medians with percentages (%) or interquartile range (IQR).

Note that the data is based on information from the entry time of the study, therefore the numbers are not representative of the general population.

The median age at inclusion was 31 years among individuals without diabetes, 51 years for individuals with type 1 diabetes, and 65 years for those with type 2 diabetes. Among individuals without diabetes, 55.4% were never married, compared with 38.0% and 12.0% among individuals with type 1 and type 2 diabetes, respectively. Furthermore, we observed disproportionate distributions of antihypertensive and cholesterol-lowering medication use across the three groups (**Tables 1**, **2**).

**Table 2:** Baseline characteristics at entry time when stratified by sex.

Characteristic	Category	Males N=3,268,516	Females N=3,290,799	Total N=6,559,315
Attained age at entry (years)	Median (IQR)	31 (18-50)	33 (18-53)	32 (18-52)
Marital status	Never married	1,911,177 (58.5%)	1,687,554 (51.3%)	3,598,731 (54.9%)
	Married or living together	1,112,722 (34.0%)	1,110,060 (33.7%)	2,222,782 (33.9%)
	Divorced or widowed	244,617 (7.5%)	493,185 (15.0%)	737,802 (11.2%)
Diabetes	No diabetes	3,213,996 (98.3%)	3,244,100 (98.6%)	6,458,096 (98.5%)
	T1DM	12,562 (0.4%)	9,919 (0.3%)	22,481 (0.3%)
	T2DM	41,958 (1.3%)	36,780 (1.1%)	78,738 (1.2%)
Insulin use	Yes	20,767 (0.6%)	17,794 (0.5%)	38,561 (0.6%)
Non-insulin use	Yes	35,146 (1.1%)	31,891 (1.0%)	67,037 (1.0%)
Cholesterol-lowering medication use	Yes	33,255 (1.0%)	24,540 (0.7%)	57,795 (0.9%)
Anti-hypertensive medication use	Yes	304,234 (9.3%)	515,802 (15.7%)	820,036 (12.5%)

Results are given in counts (n) or medians with percentages (%) or interquartile range (IQR).

Note that the data is based on information from the entry time of the study, therefore the numbers are not representative of the general population.

### 3.1 Overall tumor development

Crude analysis of the association between type 1 and type 2 diabetes and overall tumor development, including benign, pre-malignant, and malignant tumors, suggested a slightly increased hazard for individuals with both type 1 and type 2 diabetes compared with individuals without diabetes. After adjusting for covariates, HRs for individuals with type 1 diabetes moved toward no association. Adjusted HRs for individuals with type 2 diabetes suggested a slightly decreased hazard compared to individuals without diabetes (**Table 3**).

**Table 3:** Event count, event pr. 100,000 person-years, Hazard ratio (HR) with 95% confidence interval (95% CI) for overall tumor development, including benign, pre-malignant, and malignant tumors, in the type 1 diabetes group (T1DM) and in the type 2 diabetes group (T2DM), with the no diabetes group as reference.

Sex	Age	T1DM	T1DM	T1DM	T1DM	T2DM	T2DM	T2DM	T2DM
		Nb. of events	Event pr. 100,000 person-years	Crude HR (95% CI)	Multivariable model HR* (95% CI)	Nb. of events	Event pr. 100,000 person-years	Crude HR (95% CI)	Multivariable model HR* (95% CI)
Males	< 50 years	1,325	1207.21	1.09 (1.03;1.15)	1.01 (0.95;1.06)	3,135	1290.16	1.01 (0.97;1.04)	0.87 (0.84;0.90)
	> 50 years	4,577	3972.51	1.14 (1.10;1.17)	1.01 (0.99;1.04)	64,909	4119.82	1.08 (1.07;1.09)	0.96 (0.95;0.97)
Females	< 50 years	1,659	2626.88	1.08 (1.03;1.13)	1.02 (0.97;1.07)	5,924	2813.16	1.01 (0.98;1.04)	0.92 (0.89;0.94)
	> 50 years	3,308	3886.51	1.11 (1.07;1.15)	1.01 (0.98;1.05)	43,810	3737.43	1.06 (1.05;1.07)	0.96 (0.95;0.97)

*Adjusted for attained age at entry, marital status, cholesterol-lowering medication use and anti-hypertensive medication use.

### 3.2 Exploratory sub-analysis stratified on topography

**Figure 1:**
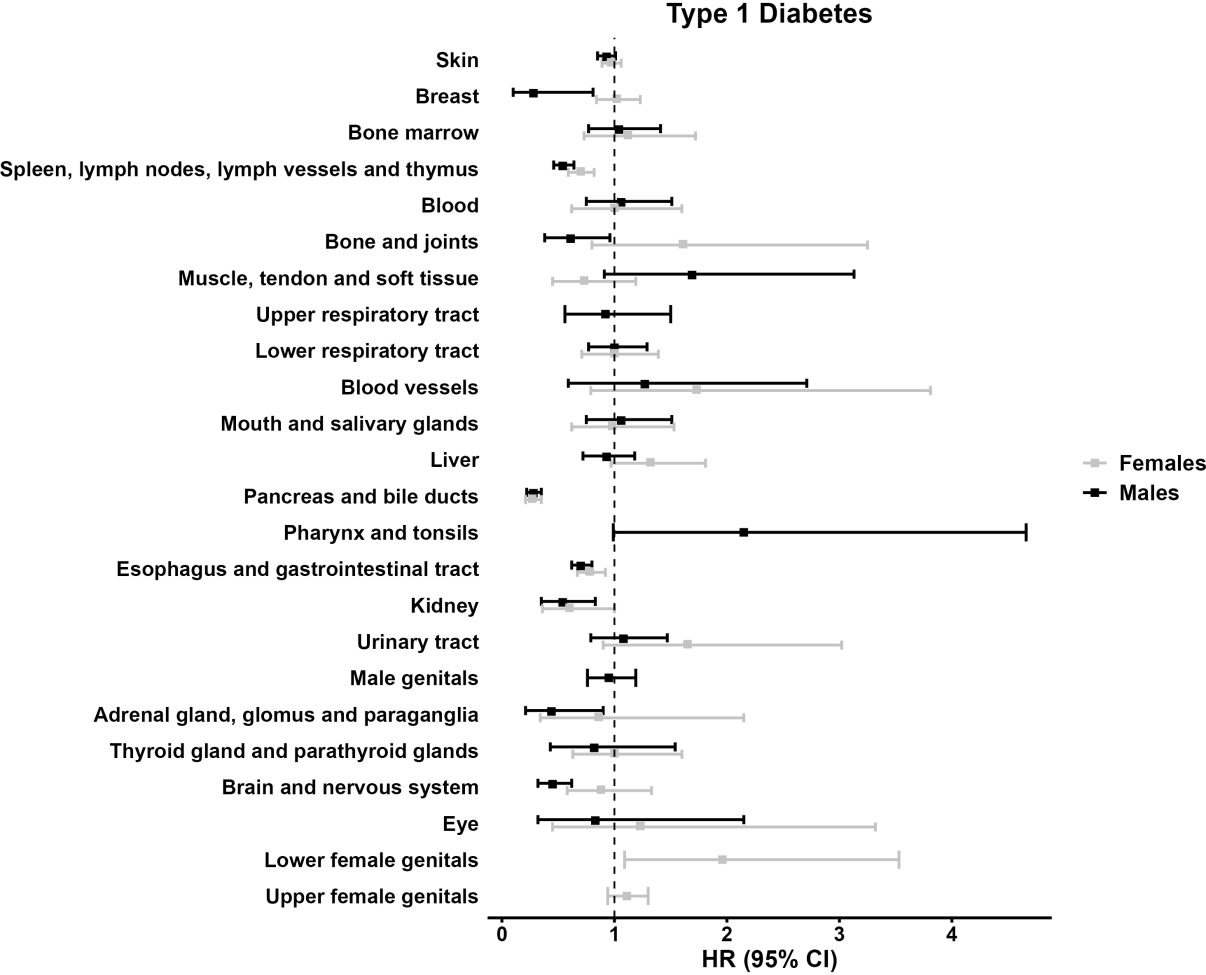
Forest plot illustrating the multivariable model hazard ratios (HR) with 95% confidence interval (95% CI) regarding the tumor development in individuals with type 1 diabetes, males, and females.

In our sub-analysis, stratified by 28 topography groups, most HRs indicated no association between type 1 diabetes and tumor development. However, we observed few substantially reduced hazards for eight topography groups, including tumors of the “pancreas and bile ducts” (HR 0.28 [0.22;0.35] among males, and HR 0.27 [0.21;0.35] among females), the “esophagus and gastrointestinal tract” (HR 0.70 [0.62;0.80] among males, and HR 0.78 [0.67;0.92] among females), and the “kidney” (HR 0.54 [0.35;0.83] among males, and HR 0.60 [0.36;1.00]). Conversely, increased hazards were rare, with only one topography group suggesting a notably increased hazard: Among females, tumors of the “lower female genitals” (HR 1.11 [1.09;3.53]) (**Table 4**, **Figure 1**).

**Table 4:** Event count, event pr. 100,000 person-years, Hazard ratio (HR) with 95% confidence interval (95% CI) for tumor development, including benign, pre-malignant, and malignant tumors, in the type 1 diabetes group (T1DM) and in the type 2 diabetes group (T2DM), with the no diabetes group as reference, stratified by tumor topography in 28 topography groups.

Tumor topography	Sex	T1DM	T1DM	T1DM	T1DM	T2DM	T2DM	T2DM	T2DM
		Nb. of events	Event pr. 100,000 person-years	Crude HR (95% CI)	Multivariable model HR* (95% CI)	Nb. of events	Event pr. 100,000 person-years	Crude HR (95% CI)	Multivariable model HR* (95% CI)
Skin	Males	2,536	1045.16	0.98 (0.90;1.07)	0.93 (0.85;1.01)	26,495	1240.54	0.94 (0.92;0.96)	0.85 (0.84;0.87)
	Females	2,325	1390.55	1.01 (0.93;1.10)	0.97 (0.89;1.06)	21,596	1272.07	0.90 (0.88;0.92)	0.81 (0.80;0.83)
Breast	Males	11	4.06	0.24 (0.10;0.61)	0.28 (0.10;0.81)	161	6.47	1.14 (0.89;1.46)	1.07 (0.81;1.42)
	Females	704	367.68	1.08 (0.90;1.29)	1.02 (0.84;1.23)	8,413	429.15	1.02 (0.99;1.06)	0.98 (0.94;1.02)
Bone marrow	Males	336	124.55	0.95 (0.71;1.26)	1.04 (0.77;1.41)	4,474	181.25	1.68 (1.58;1.77)	1.54 (1.45;1.64)
	Females	200	101.95	1.33 (0.88;2.00)	1.12 (0.73;1.72)	2,592	126.97	1.82 (1.69;1.96)	1.65 (1.52;1.79)
Spleen, lymph nodes, lymph vessels and thymus	Males	837	312.75	0.63 (0.55;0.73)	0.54 (0.46;0.64)	10,046	412.24	1.70 (1.64;1.76)	1.53 (1.47;1.60)
	Females	909	474.30	0.86 (0.74;0.99)	0.70 (0.59;0.82)	11,037	561.55	1.36 (1.32;1.40)	1.20 (1.15;1.24)
Blood	Males	258	95.52	0.99 (0.71;1.37)	1.06 (0.75;1.51)	3,504	141.67	1.65 (1.55;1.76)	1.45 (1.35;1.55)
	Females	158	80.47	1.25 (0.80;1.95)	1.00 (0.62;1.60)	2,009	98.24	1.80 (1.66;1.96)	1.58 (1.44;1.73)
Bone and joints	Males	104	38.42	0.65 (0.43;0.98)	0.61 (0.38;0.96)	1,181	47.49	2.27 (2.02;2.55)	2.21 (1.94;2.51)
	Females	79	40.16	1.90 (0.98;3.70)	1.61 (0.80;3.25)	820	39.96	1.96 (1.73;2.22)	1.64 (1.43;1.89)
Muscle, tendon and soft tissue	Males	107	39.54	1.90 (1.06;3.41)	1.69 (0.91;3.13)	1,198	48.28	1.38 (1.26;1.52)	1.19 (1.07;1.33)
	Females	86	43.80	0.84 (0.54;1.30)	0.73 (0.45;1.19)	932	45.53	1.32 (1.20;1.46)	1.15 (1.02;1.29)
Upper respiratory tract	Males	98	36.22	0.90 (0.57;1.43)	0.92 (0.56;1.50)	1,288	51.93	1.32 (1.21;1.45)	1.27 (1.15;1.41)
	Females	<5				518	25.25	1.57 (1.36;1.82)	1.41 (1.19;1.66)
Lower respiratory tract	Males	507	187.59	1.10 (0.86;1.40)	1.00 (0.77;1.29)	6,148	248.14	2.85 (2.69;3.02)	2.70 (2.53;2.87)
	Females	304	154.74	1.24 (0.91;1.69)	1.00 (0.71;1.39)	3,807	186.12	2.71 (2.53;2.90)	2.25 (2.09;2.43)
Heart	Males	<5				629	25.25	9.00 (6.69;12.13)	10.25 (7.54;13.92)
	Females	<5				370	18.00	6.99 (5.06;9.64)	9.06 (6.48;12.68)
Blood vessels	Males	58	21.40	1.20 (0.59;2.45)	1.27 (0.59;2.71)	555	22.28	5.91 (4.59;7.60)	6.52 (5.01;8.49)
	Females	54	27.42	1.43 (0.67;3.03)	1.73 (0.79;3.81)	333	16.20	5.23 (3.90;7.01)	7.14 (5.25;9.72)
Mouth and salivary glands	Males	244	90.51	1.21 (0.87;1.67)	1.06 (0.75;1.51)	2,641	107.05	1.42 (1.33;1.51)	1.26 (1.17;1.35)
	Females	130	66.34	1.10 (0.73;1.67)	0.98 (0.62;1.53)	1,668	81.85	1.21 (1.12;1.30)	1.06 (0.97;1.15)
Liver	Males	515	190.42	0.99 (0.79;1.24)	0.93 (0.72;1.18)	5,373	216.28	5.51 (5.10;5.97)	5.96 (5.48;6.48)
	Females	308	156.73	1.24 (0.92;1.67)	1.32 (0.97;1.81)	2,861	139.44	4.03 (3.68;4.41)	4.15 (3.76;4.58)
Pancreas and bile ducts	Males	336	124.45	0.33 (0.28;0.40)	0.28 (0.22;0.35)	1,885	75.82	1.91 (1.75;2.08)	1.89 (1.71;2.09)
	Females	213	108.64	0.32 (0.25;0.39)	0.27 (0.21;0.35)	1,362	66.38	1.76 (1.60;1.94)	1.64 (1.46;1.83)
Pharynx and tonsils	Males	71	26.21	1.99 (0.95;4.16)	2.15 (0.99;4.66)	879	35.36	2.30 (2.03;2.60)	2.01 (1.75;2.31)
	Females	<5				304	14.80	2.20 (1.80;2.69)	1.62 (1.29;2.04)
Esophagus and gastro-intestinal tract	Males	1,797	685.84	0.92 (0.82;1.04)	0.70 (0.62;0.80)	30,025	1311.03	1.59 (1.56;1.62)	1.13 (1.10;1.15)
	Females	1,179	618.57	1.05 (0.91;1.21)	0.78 (0.67;0.92)	17,888	929.13	1.58 (1.54;1.62)	1.15 (1.12;1.19)
Kidney	Males	132	48.78	0.79 (0.53;1.16)	0.54 (0.35;0.83)	2483	100.21	2.15 (1.99;2.33)	1.62 (1.48;1.76)
	Females	82	41.69	0.68 (0.42;1.09)	0.60 (0.36;1.00)	1182	57.67	2.26 (2.02;2.53)	1.83 (1.62;2.07)
Urinary tract	Males	350	129.95	0.96 (0.72;1.29)	1.08 (0.79;1.47)	5595	228.36	1.28 (1.22;1.34)	1.32 (1.25;1.39)
	Females	119	60.55	1.65 (0.92;2.94)	1.65 (0.90;3.02)	1449	70.80	1.46 (1.33;1.61)	1.55 (1.39;1.72)
Male genitals	Males	631	236.00	0.94 (0.76;1.16)	0.95 (0.76;1.19)	10,474	434.89	1.10 (1.07;1.14)	1.05 (1.02;1.09)
Lower female genitals	Females	127	64.69	2.13 (1.22;3.74)	1.96 (1.09;3.53)	1251	61.12	1.67 (1.52;1.84)	1.54 (1.39;1.72)
Upper female genitals	Females	860	455.77	1.16 (1.00;1.35)	1.11 (0.94;1.30)	8,713	453.43	1.09 (1.06;1.12)	1.07 (1.03;1.10)
Placenta and fetus	Females	<5				10	0.49	1.37 (0.63;3.00)	2.23 (0.96;5.18)
Pituitary gland and pineal gland	Males	<5				130	5.22	1.31 (0.99;1.72)	1.16 (0.84;1.59)
	Females	<5				107	5.21	1.96 (1.47;2.61)	1.71 (1.23;2.38)
Adrenal gland, glomus and paraganglia	Males	43	15.86	0.67 (0.36;1.26)	0.44 (0.21;0.90)	745	29.95	2.75 (2.37;3.20)	2.05 (1.74;2.43)
	Females	29	14.73	0.91 (0.39;2.11)	0.86 (0.34;2.15)	536	26.11	2.97 (2.50;3.52)	2.19 (1.80;2.65)
Thyroid gland and parathyroid glands	Males	60	22.16	0.94 (0.53;1.68)	0.82 (0.43;1.54)	719	28.93	1.40 (1.24;1.58)	1.03 (0.90;1.18)
	Females	98	49.96	0.99 (0.64;1.53)	1.00 (0.63;1.60)	1,290	63.19	1.29 (1.19;1.40)	1.05 (0.96;1.16)
Brain and nervous system	Males	178	65.82	0.55 (0.41;0.73)	0.45 (0.32;0.62)	2,200	88.80	1.48 (1.38;1.59)	1.29 (1.19;1.40)
	Females	134	68.22	0.93 (0.63;1.37)	0.88 (0.58;1.33)	1,591	77.83	1.35 (1.25;1.46)	1.07 (0.98;1.18)
Eye	Males	43	15.87	1.42 (0.59;3.40)	0.83 (0.32;2.15)	760	30.59	1.36 (1.20;1.53)	0.94 (0.82;1.08)
	Females	38	19.31	1.77 (0.69;4.58)	1.23 (0.45;3.32)	673	32.83	1.33 (1.17;1.51)	0.87 (0.76;1.00)
Ear	Males	<5				268	10.76	1.22 (0.97;1.52)	1.13 (0.88;1.45)
	Females	<5				52	2.53	1.01 (0.66;1.54)	0.82 (0.51;1.33)

*Adjusted for attained age at entry, marital status, cholesterol-lowering medication use, and anti-hypertensive medication use.

If a tumor group was only represented by 1–4 events, data were not available due to the national requirement for data protection and anonymity, and therefore, data will appear as “<5”.

**Figure 2:**
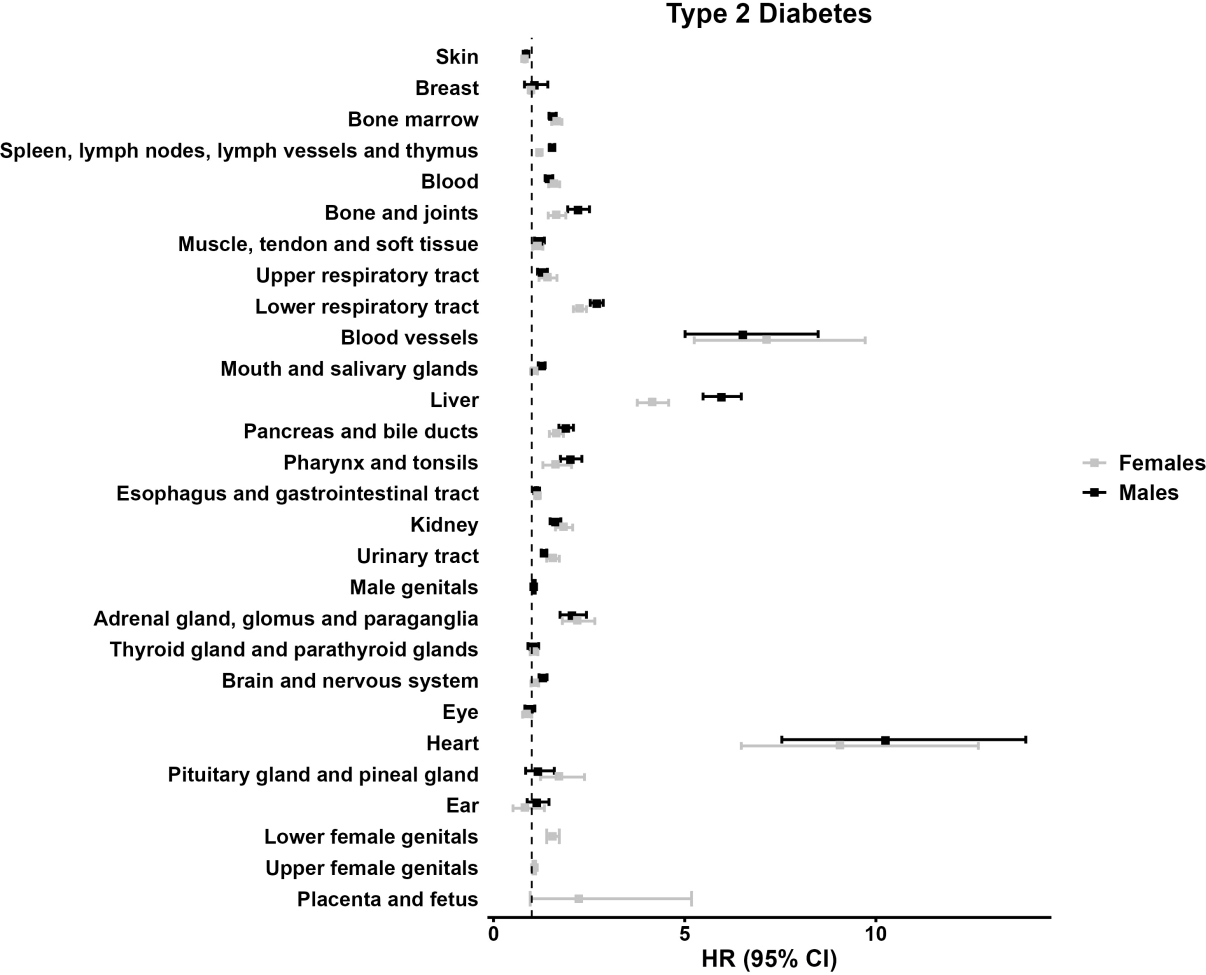
Forest plot illustrating the multivariable model hazard ratios (HR) with 95% confidence interval (95% CI) regarding the tumor development in individuals with type 2 diabetes, males, and females.

In contrast to type 1 diabetes, the sub-analysis for individuals with type 2 diabetes revealed a markedly different pattern. When stratified by 28 topography groups, 22 groups suggested increased hazards, while only one group showed a reduced hazard. The largest HRs were observed for tumors in the “heart” (HR 10.25 [7.54;13.92] among males), and HR 9.06 [6.48;12.68] among females), the “blood vessels” (HR 6.52 [5.01;8.49] among males, and HR 7.14 [5.25;9.72] among females), and the “liver” (HRs 5.96 [5.48;6.48] among males, and 4.15 [3.76;4.58] among females) (**Table 4**, **Figure 2**).

### 3.3 Supplementary analysis on overall tumor development, excluding tumors in the skin

Both crude and adjusted analyses of the association between type 1 and type 2 diabetes and overall tumor development, excluding tumors in the skin, suggested an increased hazard for individuals with both type 1 and type 2 diabetes compared with individuals without diabetes (**Table 5**).

**Table 5:** Event count, event pr. 100,000 person-years, Hazard ratio (HR) with 95% confidence interval (95% CI) for overall tumor development, including benign, pre-malignant, and malignant tumors, excluding tumors in the skin, in the type 1 diabetes group (T1DM) and in the type 2 diabetes group (T2DM), with the no diabetes group as reference.

Sex	Age	T1DM	T1DM	T1DM	T1DM	T2DM	T2DM	T2DM	T2DM
		Nb. of events	Event pr. 100,000 person-years	Crude HR (95% CI)	Multivariable model HR* (95% CI)	Nb. of events	Event pr. 100,000 person-years	Crude HR (95% CI)	Multivariable model HR* (95% CI)
Males	< 50 years	351	351.56	1.54 (1.39;1.71)	1.37 (1.23;1.52)	1138	505.88	1.45 (1.37;1.54)	1.22 (1.15;1.30)
	> 50 years	3076	2,930.10	1.28 (1.23;1.32)	1.12 (1.08;1.16)	42572	2,968.55	1.17 (1.16;1.18)	1.02 (1.01;1.03)
Females	< 50 years	540	997.54	1.36 (1.25;1.48)	1.25 (1.15;1.36)	2345	1,261.61	1.21 (1.17;1.26)	1.08 (1.03;1.12)
	> 50 years	2171	2,787.60	1.30 (1.24;1.35)	1.17 (1.12;1.22)	28223	2,623.25	1.20 (1.19;1.22)	1.07 (1.06;1.09)

*Adjusted for attained age at entry, marital status, cholesterol-lowering medication use and anti-hypertensive medication use.

## 4 Discussion

Our results suggesting no association between type 1 diabetes and overall tumor development is in contrast with existing literature, where several studies have reported increased cancer risk in the pancreas (4–6, 8, 29), the esophagus (5, 29), the stomach (29), colon (5, 8, 29), rectum (5, 8), and the kidney (3, 5, 9, 10, 30) among individuals with type 1 diabetes. Notably, our exploratory topography-specific sub-results diverge even further from prior evidence, as tumors in eight of the 28 topography groups, such as the “pancreas and bile ducts”, “esophagus and gastrointestinal tract”, and the “kidney”, consistently showed reduced hazards. However, given the potential influence of multiple testing, the hazards identified in our topographically specific sub-analysis should be interpreted with caution.

Our adjusted analyses of type 2 diabetes indicated a slightly decreased hazard on overall tumor development, which contrasts with the existing literature that consistently shows an elevated risk for several malignancies (3, 5, 9, 10, 12, 30). However, the findings from our exploratory topography-specific sub-analysis, where increased hazards were observed in 22 of 28 topography groups, aligns with existing literature on malignant tumors, such as the national Australian register-based study from 2015 (5) and the 2024 nationwide register-based cohort study from Hungary (31). This suggests that the slightly decreased hazard found for overall tumor development might be largely driven by the topography group presenting tumors in the skin, which accounted for a substantial proportion of events, underscoring the importance of sensitivity analyses excluding these tumors. We therefore conducted supplementary analyses excluding tumors in the skin. Our findings from the supplementary analyses align with those of existing literature, which supports our interpretation of the impact of skin tumors on the HRs for overall tumor development.

To our knowledge, no previous studies have examined the association between diabetes and the topography groups, suggesting the largest HRs observed in this study (tumors in the “heart” and the “blood vessels”). While estimates from our exploratory analyses should be interpreted with caution, our findings warrant further exploration with a focus on these sub-groups. Even though tumors of the heart are a very rare condition, the (obstructive) consequences might be severe due to valve dysfunction, arrhythmias, etc. (32).

### 4.1 Correlation, competing risks, and reverse causality

Beyond the exclusion of tumors in the skin, the differences between overall tumor development and our stratified analyses can reflect methodological choices. First, because each stratified analysis considered only the first tumor per topography, individuals developing tumors across multiple locations could therefore appear in several sub-analyses. This can introduce correlation between strata and may artificially elevate HRs in stratified analyses where frequent tumor recurrences concentrate in high-risk individuals.

Second, competing risks are a general concern in studies regarding the association between diabetes and cancer, potentially biasing estimates if not addressed. Bjornsdottir et al., 2020 have shown the impact of this competing risk, by doing a competing risk analysis using Fine-Gray, including lung cancer and death from any cause as the two competing events. Their estimated sub-distributed hazard ratio for lung cancer comparing individuals with and without type 2 diabetes failed to reach statistical significance after taking death into account (30). While our study did not include competing-risk analyses, this example illustrates that competing events can inflate our cause-specific HRs.

Additionally, reverse causality represents another important consideration, particularly for tumors of the pancreas and liver, and potentially other sites (5, 33). Previous studies have suggested that dysfunction in insulin regulation and secretion may not only precede tumor development but could also occur as a direct consequence of tumor growth, complicating causal interpretation (5, 10, 33). Incorporating strategies to address reverse causality, such as lag-time analyses or sensitivity analyses excluding early follow-up, would therefore be essential in future research to disentangle these bidirectional effects.

Furthermore, though we believe inclusion of benign and pre-malignant tumors provides a more comprehensive estimate of tumor burden, direct comparison with existing literature is challenging, as most prior research focused exclusively on malignancies. The inclusion of benign and pre-malignant tumors in our study may partially explain the observed discrepancies. Additionally, tumor classification using SNOMED codes in the present study further differed from tumor classification in existing literature, which mostly used ICD codes of cancer diagnosis.

### 4.2 Underlying mechanisms

Several biological mechanisms have been discussed to explain the underlying mechanisms of the association between diabetes and tumors, such as increased inflammation status, increased oxidative stress, obesity, hyperglycemia, and hyperinsulinemia (3, 10, 34–36). Multiple studies have evaluated the cancer risk related to the administration of exogenous insulin and other diabetic medications. A 2023 review and meta-analysis found that insulin (and insulin secretagogue) use was associated with an increased pancreatic cancer risk (37), while the use of biguanide and thiazolidinedione (type 2 diabetes medication) carried no risk, or potentially a lower risk of some cancers (37–39). It is unclear to what extent these mechanisms affect the association of benign and pre-malignant tumors. Therefore, adjustment regarding the use of different types of diabetes medication would be preferable to distinguish between the effect of medication and the underlying diabetes disease.

### 4.3 Challenges of unmeasured confounding

A key limitation of this study is the lack of available data on lifestyle-related confounders such as smoking, diet, physical activity, and alcohol consumption. These lifestyle factors are well-documented determinants of cancer risk and may differ systematically between individuals with and without type 1 and type 2 diabetes (33), highlighting the potential for confounding bias in our estimated HRs. The inability to adjust for these unmeasured confounders could partially explain some of the observed associations. Shin et al., 2024 investigated the association between type 1 diabetes and risk of developing gastrointestinal cancers in a large cohort, adjusted for both tobacco use, body mass index, physical activity, household income, and alcohol consumption. Direct comparison of estimates between studies is challenging due to differences in study design, covariate adjustment, and population characteristics, however, their estimates all move toward no association from their unadjusted to adjusted analyses (29). It is plausible that our estimates are biased away from the null due to unmeasured confounding, as suggested by those findings. Likewise, potential bias regarding human papillomavirus (HPV) vaccination status and cancer screening procedures (10) was not taken into account. However, our analyses provide population-level estimates that reflect real-world clinical tumor patterns, offering valuable insights into the overall tumor burden among individuals with diabetes in a nationwide population.

To assess the robustness of our estimates, we calculated E-values, which reflect the minimum strength of association that an unmeasured confounder would need to fully explain the observed association (40). The calculated E-values are presented in the **Supplementary Material (Appendix 3)**. For the largest HRs observed, regarding tumors of the “heart” and “blood vessels” (HRs 10.25 [7.54;13.92] (males, heart) and 7.14 [5.25;9.72] (females, blood vessels)), corresponding E-values were 19.99 and 13.76, respectively. This indicates that any confounder would need to be strongly associated with both diabetes and these tumors to account for the observed associations.

### 4.4 Assessing the role of detection bias

Detection bias is an acknowledged concern in studies of diabetes and tumor development, as individuals with diabetes typically have more frequent healthcare encounters, potentially increasing incidental detection of certain tumors (5, 33). Such bias is likely tumor-specific and depends on whether diabetes care includes targeted investigations of the relevant organ system. For example, routine diabetes management does not include systematic cardiac imaging or other diagnostics that would specifically increase the detection of cardiac tumors. In contrast, organ systems commonly monitored in diabetes care, such as the eyes, may be more susceptible to detection bias due to regular examinations. Although the magnitude of this bias may vary, evidence suggests that inflation of cancer detection is mainly concentrated around the time of diabetes diagnosis, as shown in lag-period analyses (41), which may indicate an inflation of our estimated HRs. However, in our study, many subgroup analyses suggested reduced hazard ratios for tumor development in individuals with type 1 diabetes, indicating that any potential detection bias is unlikely to have substantially influenced risk estimates in this group. Nevertheless, some degree of detection bias cannot be excluded and should be considered when interpreting the associations observed, and therefore the findings should be interpreted with caution.

### 4.5 Data quality

In terms of register-based healthcare research, Denmark is recognized to provide validated, high-quality data at an individual level for nearly 6 million inhabitants (24, 26). The present study provided a long-term follow-up time and a large sample size. This is highly preferred, especially when studying site-specific tumors, due to the low numbers of events in some tumor groups. Over a period of 23 years, 128,647 tumor events were observed in the diabetes patients, 10,869 in the type 1 diabetes patients, and 117,788 in the type 2 diabetes patients.

Type 1 and type 2 diabetes were thoroughly distinguished in the data and presentation of the results throughout this paper, due to the different etiology and pathophysiology of the diseases. By combining comprehensive nationwide coverage, a long observation period, and precise differentiation between diabetes types, this study provides robust population-level evidence on the long-term endocrine-metabolic links between diabetes and tumor development. However, the diabetes classification algorithm relies on registry data, which inherently introduces a degree of uncertainty. Data from the Danish National Prescription Registry is only available from 1995 onward. Registry coding errors can occur, and our model does not account for alternative diabetes types such as latent autoimmune diabetes in adults (LADA) or maturity-onset diabetes of the young (MODY).

Data on SNOMED codes were likewise considered high quality with minimal risk of errors or misclassification, as all SNOMED codes are based on biopsy-verified diagnoses (24), which represents a substantial strength of this study. Furthermore, with tumor categories based on SNOMED topography codes, it will be possible to further study each tumor group, by stratifying into categories of pathology details in future research. This will be valuable in the process of understanding the underlying mechanisms regarding the association between diabetes and tumor biology.

### 4.6 Conclusion

This nationwide register-based cohort study identified significant associations between diabetes and tumor development, with variation observed across tumor sites, sex, and diabetes type. To our knowledge, this is the first study to provide a comprehensive description of general tumor burden, including benign, pre-malignant, and malignant tumors, among individuals with diabetes. These associations are clinically relevant, given the potential health consequences of tumor growth, obstruction, and malignancy. Collectively, our findings contribute to observational evidence and further hypotheses development regarding the complex metabolic and endocrine processes potentially involved in the relationship between diabetes and tumor development. Future research should leverage the detailed SNOMED-based tumor classifications available in Danish health registries to further examine pathological subtypes and site-specific patterns in greater depth, thereby improving our understanding of possible links between diabetes and tumor biology across different tissues.

## Data Availability

The data underlying this study are held by the Danish Health Data Authority and are not publicly available due to legal and privacy restrictions. Access to these data can be requested from the Danish Health Data Authority for researchers meeting their criteria for access. All procedures comply with Danish data protection regulations.
