# Supplementary material for: Diabetes and tumor risk: a 23-year Danish national cohort study
Source: Front Endocrinol (Lausanne). 2026 Jan 7;16:1725065. doi: 10.3389/fendo.2025.1725065 (PMC12819280; doi:10.3389/fendo.2025.1725065)
Supplement: Supplementary file 1 [file Table1.docx]

***Supplementary material***

**Appendix 1. Categorization of type 1 and type 2 diabetes.**

| Type 1 diabetes |
| --- |
| Diagnostic code E10*  AND  First prescription of A10A* within a year of first E10* diagnosis  AND  Last prescription of A10A* within a year of exit  AND  Number of prescriptions ≥ number of years from first prescription to exit |

| Type 2 diabetes | Excluded |
| --- | --- |
| Diagnostic code E11*  AND  ≥ two A10B* prescriptions** | Already grouped as type 1 diabetes  AND/OR  Female AND diagnostic code for PCOS (E282) AND no diagnostic code for diabetes type II (E11*) |
| OR |  |
| ≥ two E11* diagnostic codes |  |
| OR |  |
| ≥ two prescriptions of A10A* if age 40+ at prescription |  |
| OR |  |
| ≥ two prescriptions of A10B* if age 30+ at prescription |  |

Full description of codes can be found in Appendix 2

*All subgroups are included in this code

**One prescription is allowed for patients who received their diagnosis during 2022.

**Appendix 2. Full list of the SNOMED T-codes related to each tumor group, SNOMED M-codes, ICD codes, and ATC codes used in this study.**

**SNOMED T-codes**

| Topography | SNOMED T-codes |
| --- | --- |
| Skin | T01*, T02*, T03* |
| Breast | T04* |
| Bone marrow | T06* |
| Spleen, lymph nodes, lymph vessels and thymus | T07*, T08*, T09*, T98* |
| Blood | T0x* |
| Bone and joints | T10*, T11*, T12*, T1x5*, T1x7* |
| Muscle, tendon and soft tissue | T13*, T14*, T16*, T17*, T18*, T1x0*, T1x2*, T1x3* |
| Upper respiratory tract | T21*, T22*, T23*, T24* |
| Lower respiratory tract | T25*, T26*, T27*, T28*, T29* |
| Heart | T31*, T32*, T33*, T34*, T35*, T36*, T37*, T38*, T39* |
| Blood vessels | T40*, T41*, T42*, T43*, T44*, T45*, T46*, T47*, T48*, T49* |
| Mouth and salivary glands | T51*, T52*, T53*, T54*, T55* |
| Liver | T56* |
| Pancreas and bile ducts | T57*, T58*, T59*, T99* |
| Pharynx and tonsils | T60*, T61* |
| Esophagus and gastrointestinal tract | T62*, T63*, T64*, T65*, T66*, T67*, T68*, T69* |
| Kidney | T71* |
| Urinary tract | T72*, T73*, T74*, T75* |
| Male genitals | T76*, T77*, T78*, T79* |
| Lower female genitals | T80*, T81* |
| Upper female genitals | T82*, T83*, T84*, T85*, T86*, T87* |
| Placenta and fetus | T88*, T89* |
| Pituitary gland and pineal gland | T91*, T92* |
| Adrenal gland, glomus and paraganglia | T93*, T94*, T95* |
| Thyroid gland and parathyroid glands | T96*, T97* |
| Brain and nervous system | Tx0*, Tx1*, Tx2*, Tx3*, Tx4*, Tx5*, Tx6*, Tx7*, Tx8*, Tx9* |
| Eye | Txx* |
| Ear | Txy* |

*All codes after the star (*) were included in the specific topography group.

**SNOMED M-codes**

| Neoplasia/tumor | M8-, M9- |
| --- | --- |

**ICD codes**

| E10* | Type 1 diabetes |
| --- | --- |
| E11* | Type 2 diabetes |
| E282 | PCOS |

*All codes after the star (*) were included in the specific ICD code.

**ATC codes**

| A10A* | Insulin and analogues |
| --- | --- |
| A10B* | Blood glucose-lowering drugs, excluding insulin |
| C03*, C07*, C08*, C09* | Anti-hypertensive medication |
| C10* | Cholesterol-lowering medication |

*All codes after the star (*) were included in the specific ATC code.

**Appendix 3. E-values on selected analyses, in the type 1 diabetes group (T1DM) and in the type 2 diabetes group (T2DM).**

| Tumors topography |  | E-value T1DM | E-value T2DM |
| --- | --- | --- | --- |
| Skin | Males | 1.36 (1) | 1.63 (1.56) |
|  | Females | 1.21 (1) | 1.77 (1.7) |
| Heart | Males | - | 19.99 (14.56) |
|  | Females | - | 17.61 (12.44) |
| Blood vessels | Males | 1.86 (1) | 12.52 (9.49) |
|  | Females | 2.85 (1) | 13.76 (9.97) |
| Liver | Males | 1.36 (1) | 11.4 (10.43) |
|  | Females | 1.95 (1) | 7.77 (6.98) |
| Pancreas and bile ducts | Males | 6.6 (5.16) | 3.19 (2.81) |
|  | Females | 6.87 (5.16) | 2.66 (2.28) |
| Overall tumor development, excluding tumors in the skin |  |  |  |
| Males | < 50 years | 2.08 (1.76) | 1.74 (1.57) |
|  | > 50 years | 1.49 (1.37) | 1.16 (1.11) |
| Females | < 50 years | 1.81 (1.57) | 1.37 (1.21) |
|  | > 50 years | 1.62 (1.49) | 1.34 (1.31) |

All E-values are calculated using: https://www.evalue-calculator.com/evalue/
